# Incidence of SARS-CoV-2 Infection Among Health Care Personnel, First Responders, and Other Essential Workers During a Prevaccination COVID-19 Surge in Arizona

**DOI:** 10.1001/jamahealthforum.2021.3318

**Published:** 2021-10-22

**Authors:** Katherine D. Ellingson, Joe K. Gerald, Xiaoxiao Sun, James Hollister, Karen Lutrick, Joel Parker, Patrick Rivers, Shawn C. Beitel, Zoe Baccam, Julie Mayo Lamberte, Lauren Grant, Elizabeth Kim, Rachana Bhattarai, Kenneth Komatsu, Jennifer Meece, Preeta K. Kutty, Mark G. Thompson, Jefferey L. Burgess

**Affiliations:** 1 Department of Epidemiology and Biostatistics, Zuckerman College of Public Health, University of Arizona, Tucson; 2 Department of Community, Environment and Policy, University of Arizona, Tucson; 3 Department of Family and Community Medicine, University of Arizona, Tucson; 4 Department of Health Promotion Sciences, University of Arizona, Tucson; 5 US Centers for Disease Control and Prevention, Atlanta, Georgia; 6 Arizona Department of Health Services, Phoenix; 7 Marshfield Laboratories, Marshfield, Wisconsin

## Abstract

**Importance:**

Understanding the relative risk of SARS-CoV-2 infection across occupations can inform guidance to protect workers and communities. Less is known about infection risk for first responders and other essential workers than for health care personnel.

**Objective:**

To compare the prevaccination incidence of SARS-CoV-2 infection among first responders and other essential workers with incidence among health care personnel.

**Design, Setting, and Participants:**

This was a prospective cohort study of health care personnel, first responders, and other essential workers in Arizona from July 20, 2020, to March 14, 2021. Participants were seronegative at enrollment, had frequent direct contact with others at work, worked at least 20 hours per week, and submitted weekly nasal swab specimens for real-time reverse transcriptase polymerase chain reaction analysis. Data analyses were performed from April 19, 2021, to June 4, 2021.

**Exposures:**

Occupation was the primary exposure of interest. Confounders assessed were sociodemographic characteristics, health status, community exposure, and work exposure.

**Main Outcomes and Measures:**

Crude incidence of SARS-CoV-2 infection was defined as the sum of first positive SARS-CoV-2 infections in participants divided by person-weeks at risk. Negative binomial regression was used to model SARS-CoV-2 infection by occupation to estimate unadjusted and adjusted incidence rate ratios (IRRs). The least absolute shrinkage and selection operator (LASSO) method was used to generate a parsimonious multivariable model.

**Results:**

The study cohort comprised 1766 Arizona workers (mean age [SD], 43.8 [11.1] years; 1093 [61.9%] female; 401 [22.7%] were Hispanic and 1530 [86.6%] were White individuals) of whom 44.2% were health care personnel, 22.4% first responders, and 33.4% other essential workers. The cohort was followed up for 23 393 person-weeks. Crude incidence of SARS-CoV-2 infection was 6.7, 13.2, and 7.4 per 1000 person-weeks at risk for health care personnel, first responders, and other essential workers, respectively. In unadjusted models, first responders had twice the incidence of infection as health care personnel (IRRs, 2.01; 95% CI, 1.44-2.79). While attenuated, this risk remained elevated in adjusted LASSO-optimized models (IRR, 1.60; 95% CI, 1.07-2.38). Risk of infection among other essential workers was no different than for health care personnel in unadjusted or adjusted models.

**Conclusions and Relevance:**

This prospective cohort study found that first responders had a higher incidence of SARS-CoV-2 infection than health care personnel, even after adjusting for potential confounding factors. Given their frequent contact with each other and with the public and their high rates of SARS-CoV-2 infection, the safety challenges for first responders warrant greater public health attention and research.

## Key Points

**Question:**

Before COVID-19 vaccine availability, how comparable were rates of SARS-CoV-2 infection among health care personnel, first responders, and other essential workers?

**Findings:**

This prospective cohort study of 1766 unvaccinated seronegative Arizona workers using self-administered reverse-transcription polymerase chain reaction testing found that first responders had a significantly higher incidence of SARS-CoV-2 infection than health care personnel, even after controlling for sociodemographic characteristics and underlying health and exposure indicators.

**Meaning:**

The findings of this cohort study indicate that first responders warrant greater public health attention in context of the COVID-19 pandemic given their higher rates of SARS-CoV-2 infection.

## Teaser

This prospective cohort study compares the prevaccination incidence of SARS-CoV-2 infection among first responders and other essential workers with incidence among health care personnel in Arizona before the availability of a COVID-19 vaccine.

## Introduction

The COVID-19 pandemic has created unprecedented occupational safety challenges.^1^ The first globally reported cases of COVID-19, caused by SARS-CoV-2, were among health care personnel and other occupations requiring frequent and direct contact with others.^2^ In the US, tens of millions of workers are employed in positions that expose them to contact with others who might harbor transmissible infectious diseases.^3^ Understanding the relative risk of SARS-CoV-2 infection by occupation can inform mitigation efforts to protect workers and their constituent communities.

The increased risk of SARS-CoV-2 infection among health care personnel has been well established.^4,5,6^ Risk levels and factors associated with infection among nonhealth care occupations are less thoroughly understood. First responders have frequent direct contact with the public and coworkers. They also frequently interact with patients and individuals living in congregate settings that typically have less intensive infection prevention protections than health care facilities. Similarly, other essential workers may be at increased risk of SARS-CoV-2 infection owing to fewer infection prevention protocols and other unexplored risk factors related to their interaction with each other or the general public. Our objective was to compare the prevaccination incidence of SARS-CoV-2 infection among first responders and other essential workers with incidence among health care personnel in a statewide prospective cohort during Arizona’s largest surge in COVID-19 cases.^7^

## Methods

### Study Design

The Arizona Healthcare, Emergency Response, and Other Essential Workers Study (AZ HEROES) is a statewide prospective cohort formed to examine SARS-CoV-2 infection and immunity among frontline and essential workers. This prospective cohort study was reviewed and approved by the institutional review board (IRB) of the University of Arizona as the single IRB. Informed consent was obtained from eligible and interested participants via an electronic consent form reviewed and signed electronically through REDCap.^8^ All methods and outcomes are reported according to the best practices described in the Strengthening the Reporting of Observational Studies in Epidemiology (STROBE) Statement reporting guidelines.^9^

### Participants, Recruitment, and Retention

Recruitment began in July 2020 with target enrollment set for 2000 seronegative and 2000 seropositive participants. Sampling targets included 40% health care personnel, 30% first responders, and 30% other essential workers. Similarly, investigators sought to enroll 50% female, and achieve race and ethnicity distributions commensurate with Arizona’s population. Recruitment included outreach to participants in a statewide antibody testing initiative targeting essential workers, who agreed to be contacted for future research studies. In addition, print, online, and social media advertisements were broadly distributed in the public domain. Participants were screened for eligibility by telephone or through an online self-screening questionnaire. Participant support coordinators were employed to enhance retention by keeping lines of communication open, maintaining frequent follow-up with participants, answering logistical questions by telephone or email, and referring questions requiring subject-matter expertise to senior study investigators. Coordinators also produced monthly newsletters in which informational updates and study findings were highlighted. Beginning in February 2021, monetary incentives—$50 gift card drawings—were offered to participants who consistently submitted required specimens and surveillance data.

### Inclusion and Exclusion Criteria

Participants were Arizona residents aged 18 to 85 years, who worked at least 20 hours per week in occupations involving regular direct contact (within 3 feet) with others. For the purpose of this study, “health care personnel” included only those workers providing direct patient care at inpatient, outpatient, and residential health care settings. Per US Department of Labor standards,^10^ the category “first responders” comprised correctional officers, fire fighters, law enforcement (including US Customs and Border Protection), and nonfire emergency medical services (EMS) workers. The category “other essential workers” included all other individuals in work environments that precluded physical distancing and subcategories that included frontline education, childcare or social work, frontline retail or hospitality, essential operations in government and nonprofit sectors requiring in-person work (eg, postal workers or 911 call center operators), and essential infrastructure workers (eg, waste management, agriculture, utility services).

Participants submitted nasal specimens on a weekly basis using home testing kits and reported any symptoms of COVID-like illness on a weekly basis via text message on a mobile device. Self-collection home test kits included nasal swabs for weekly surveillance, as well as saliva specimen collection kits, which were to be used in addition to a nasal swab at the onset of any symptoms of COVID-like illness. All specimens were sent for testing via real-time reverse transcription polymerase chain reaction (RT-PCR) assay at the Marshfield Clinic Research Institute (Marshfield, Wisconsin).

For this analysis, only potential participants who were seronegative at enrollment were included; those who were seropositive or of unknown serostatus at enrollment were excluded. Participants who were less than 80% compliant with weekly specimen submissions were excluded. Additional details on the AZ HEROES protocols are published elsewhere.^11^

### Outcomes, Risk Factors, and Confounders

Crude incidence of SARS-CoV-2 infection was calculated by dividing the number of incident infections by person-weeks at risk; incident infections included positive RT-PCR results from participants who had not had a previous positive test result. Person-time was calculated from participants’ date of enrollment through March 14, 2021, and was censored at the date of specimen collection for a positive RT-PCR test or date of first vaccination.

Occupational category was the primary independent variable. Confounders included demographic characteristics, socioeconomic factors, underlying health status, community exposure, and work exposure (Table 1). Participant age, race and ethnicity, and sex were self-reported at screening or on enrollment surveys. Socioeconomic variables—household size, household income, and highest level of education attained—were self-reported at enrollment, as was self-reported health at baseline and the presence of 1 or more chronic health conditions. Work exposure was characterized by the percentage of time that personal protective equipment (PPE) was worn at work as directed by the participant’s employer, mean number of total hours worked per week, and mean number of hours per week worked within 3 feet of other individuals. Community exposure was measured by the percentage of time a mask was worn while in public but not at work. To account for variable population-based exposure to SARS-CoV-2 throughout the study period, an aggregate community exposure measure was calculated for each participant by averaging the county-specific weekly incidence per 100 000 population over the person-weeks of participation.

**Table 1.** Characteristics of Participants in the AZ HEROES Cohort Who Were SARS-CoV-2 Seronegative at Enrollment and Contributed at Least 1 Person-Week of Data, July 20, 2020, through March 14, 2021

Characteristics	Occupation, No. (%)			
	Health care personnel (n = 781)	First responders (n = 395)	Other essential workers (n = 590)	Total (n = 1766)
Outcomes				
Incident SARS-CoV-2	75 (9.6)	68 (17.2)	52 (8.8)	195 (11.0)
Person-weeks, median (IQR)	15.0 (8.7-18.7)	12.9 (6.6-18.0)	11.9 (9.3-14.7)	12.9 (8.6-17.4)
% Compliance, median (IQR)	100 (96.3-100)	100 (93.8-100)	100 (100-100)	100 (96.2-100)
Demographics				
Sex				
Male	233 (29.8)	248 (62.8)	182 (30.8)	663 (37.5)
Female	544 (69.7)	146 (37.0)	403 (68.3)	1093 (61.9)
Other^a^	2 (0.3)	0 (0.0)	3 (0.5)	5 (0.3)
Missing data	2 (0.3)	1 (0.3)	2 (0.3)	5 (0.3)
Race				
African American/Black	4 (0.5)	9 (2.3)	6 (1.0)	19 (1.1)
American Indian/Alaska Native	5 (0.6)	5 (1.3)	2 (0.3)	12 (0.7)
Asian	31 (4.0)	2 (0.5)	9 (1.5)	42 (2.4)
White	675 (86.4)	335 (84.8)	520 (88.1)	1530 (86.6)
Multiracial	48 (6.1)	30 (7.6)	44 (7.5)	122 (6.9)
Did not disclose	18 (2.3)	14 (3.5)	9 (1.5)	41 (2.3)
Missing data	0 (0.0)	0 (0.0)	0 (0.0)	0 (0.0)
Ethnicity				
Hispanic	178 (22.8)	102 (25.8)	121 (20.5)	401 (22.7)
Non–Hispanic	586 (75.0)	283 (71.6)	455 (77.1)	1324 (75.0)
Did not disclose	13 (1.7)	7 (1.8)	11 (1.9)	31 (1.8)
Missing data	4 (0.5)	3 (0.8)	3 (0.5)	10 (0.6)
Age, mean (SD)	43.1 (11.1)	42.8 (9.5)	46.8 (11.7)	43.8 (11.1)
Socioeconomic status				
Household size, mean (SD)	3.1 (1.5)	3.4 (1.6)	3.0 (1.4)	3.2 (1.5)
Missing data	42 (5.4)	20 (5.1)	27 (4.6)	89 (5.0)
Household income, $				
<49 999	39 (5.0)	26 (6.6)	87 (14.7)	152 (8.6)
50 000-99 999	212 (27.1)	143 (36.2)	224 (38.0)	579 (32.8)
100 000-199 999	294 (37.6)	186 (47.1)	201 (34.1)	681 (38.6)
≥200 000	195 (25.0)	23 (5.8)	41 (6.9)	259 (14.7)
Missing data	41 (5.2)	17 (4.3)	37 (6.3)	95 (5.4)
Education				
High school or less	2 (0.3)	12 (3.0)	19 (3.2)	33 (1.9)
Some college	47 (6.0)	108 (27.3)	94 (15.9)	249 (14.1)
College degree or higher	699 (89.5)	259 (65.6)	454 (76.9)	1412 (80.0)
Missing	33 (4.2)	16 (4.1)	23 (3.9)	72 (4.1)
Health				
≥1 Chronic condition	258 (33.0)	113 (28.6)	203 (34.4)	574 (32.5)
Self-rated health, mean (SD)^b^	4.07 (0.8)	3.95 (0.8)	3.87 (0.8)	3.97 (0.8)
Work exposure, mean (SD)				
% Time PPE worn as directed^c^	91.6 (12.3)	71.1 (25.3)	90.8 (15.8)	86.8 (19.0)
Hours worked per week	41.4 (11.4)	54.5 (18.4)	41.3 (9.4)	44.3 (13.9)
Hours worked within 3 ft of others	30.6 (13.8)	29.5 (17.7)	20.3 (13.6)	26.9 (15.4)
Community exposure, mean (SD)				
County incidence during study^d^	5.83 (0.37)	5.87 (0.41)	5.89 (0.45)	5.86 (0.41)
% Time mask worn in public^e^	90.8 (13.6)	85.5 (20.7)	91.0 (15.2)	89.6 (15.8)

Abbreviations: AZ HEROES, Arizona Healthcare, Emergency Response, and Other Essential Workers Study; PPE, personal protective equipment.

^a^ Other sex includes transgender, non–gender-conforming, and preferred not to answer.

^b^ Measured on a 5-point scale, from 1 (worst) to 5 (best).

^c^ At work, percentage of time PPE worn per employer’s requirements.

^d^ County-level COVID-19 incidence per 100 000 population averaged over each participant’s weeks in the study.

^e^ Not at work, percentage of time a mask worn in public (eg, running errands).

### Statistical Analysis

Incidence was modeled with individual-level negative binomial regression. Nonindependence of observations by geographic area of residence was addressed by adding random intercepts by county. Incidence rate ratios (IRRs) for SARS-CoV-2 infection were estimated by occupation in an unadjusted model and in several models that adjusted for grouped confounders as follows: demographics, socioeconomic status, underlying health status, community exposure, and work exposure (Table 2). Finally, a full model was specified with all variables included, as well as a parsimonious model created using the least absolute shrinkage and selection operator (LASSO) method for variable selection.^12^ Unadjusted and adjusted IRRs and 95% CIs are presented. Statistical tests were 2-tailed and *P* values of < .05 were considered statistically significant. Data analyses were performed from April 19, 2021, to June 4, 2021, using R, version 4.0.4 (The R Foundation for Statistical Computing) and SAS, version 9.4 (SAS Institute Inc).

**Table 2.** Results of Multivariable Negative Binomial Regression Model of SARS-CoV-2 Infection Incidence, by Occupation, in Unadjusted Models and Models Adjusted for Demographics, Community Risk, Social Factors, and Race and Ethnicity for the AZ HEROES Cohort, July 20, 2020, through March 14, 2021

Factors	Incidence rate ratio (95% CI)							
	Unadjusted	Demographic information only	Socioeconomic status only	Underlying health status only	Adjustment factors			
					Work exposure only	Community exposure only	Fully adjusted model	LASSO optimization
Occupation								
First responder (reference, health care personnel)	2.01 (1.44-2.79)	1.75 (1.22-2.50)	1.81 (1.24-2.64)	2.16 (1.54-3.05)	1.99 (1.32-3.00)	1.96 (1.39-2.76)	1.63 (1.04-2.55)	1.60 (1.07-2.38)
Other essential worker (reference, health care personnel)	1.10 (0.77-1.57)	1.18 (0.82-1.70)	1.06 (0.72-1.55)	1.17 (0.81-1.69)	1.18 (0.81-1.72)	1.05 (0.72-2.76)	1.02 (0.67-1.56)	1.06 (0.71-1.58)
Demographic information								
Non–White race (White, reference)	NA	1.40 (0.91-2.17)	NA	NA	NA	NA	1.31 (0.81-2.11)	1.31 (0.81-2.12)
Hispanic ethnicity (non–Hispanic, reference)	NA	1.27 (0.90-1.79)	NA	NA	NA	NA	1.25 (0.87-1.81)	1.26 (0.87-1.81)
Sex (female, reference)	NA	1.37 (1.01-1.86)	NA	NA	NA	NA	1.38 (0.98-1.94)	1.38 (0.99-1.91)
Age^a^	NA	0.81 (0.70-0.93)	NA	NA	NA	NA	0.81 (0.69-0.95)	0.81 (0.70-0.95)
Socioeconomic status								
Income ≥$50 000 (<$50 000, reference)	NA	NA	0.77 (0.45-1.33)	NA	NA	NA	0.89 (0.50-1.56)	0.89 (0.51-1.56)
Education (college degree or higher, reference)	NA	NA	1.35 (0.91-2.00)	NA	NA	NA	1.33 (0.88-1.99)	1.30 (0.87-1.94)
Household size^b^	NA	NA	1.07 (0.97-119)	NA	NA	NA	1.07 (0.96-1.18)	1.06 (0.96-1.17)
Underlying health status								
≥1 Chronic condition (0, reference)	NA	NA	NA	0.98 (0.72-1.33)	NA	NA	1.22 (0.87-1.71)	1.22 (0.88-1.70)
Self-rated health^c^	NA	NA	NA	0.93 (0.77-1.11)	NA	NA	0.99 (0.81-1.21)	Dropped^h^
Work exposures								
% Time PPE worn as directed^d^	NA	NA	NA	NA	0.98 (0.91-1.06)	NA	1.02 (0.94-1.12)	Dropped^h^
Hours worked per week^e^	NA	NA	NA	NA	0.99 (0.88-1.12)	NA	1.01 (0.88-1.16)	Dropped^h^
Hours worked within 3 ft of others^e^	NA	NA	NA	NA	1.03 (0.93-1.15)	NA	0.97 (0.86-1.09)	Dropped^h^
Community exposures								
County incidence during study^f^	NA	NA	NA	NA	NA	1.84 (0.93-3.57)	1.90 (0.95-3.80)	1.92 (0.96-3.82)
% Time mask worn in public^g^	NA	NA	NA	NA	NA	0.94 (0.87-1.03)	0.94 (0.86-1.04)	0.95 (0.87-1.04)

Abbreviations: AZ HEROES, Arizona Healthcare, Emergency Response, and Other Essential Workers Study; IRR, incidence rate ratio; LASSO, least absolute shrinkage and selection operator; NA, not applicable; PPE, personal protective equipment.

^a^ IRR reported per 10-year increase in age.

^b^ IRR reported per 1-unit increase in household size.

^c^ IRR reported per 1-unit increase on a 5-point scale (1 = worst to 5 = best).

^d^ IRR per 10% increase in time that PPE worn at work per employer’s requirements.

^e^ IRR per 10-hour increase in hours worked or in close contact with others per week.

^f^ IRR per 10-unit change in county-level incidence per 100 000 population averaged for weeks in the study.

^g^ IRR per 10% increase in time masks worn when not working but in public spaces (eg, running errands).

^h^ Variable dropped with LASSO optimization method.

## Results

From July 20, 2020, to March 14, 2021, a total of 1901 seronegative participants were enrolled; 135 (7.1%) were excluded for low compliance with specimen collection. The analytic sample comprised 1766 participants (mean age [SD], 43.8 [11.1] years; 1093 [61.9%] female) of whom 401 (22.7%) were Hispanic and 1530 (86.6%) were White individuals (Table 1). The sample was followed up for 23 393 person-weeks at risk. Occupational class included 781 (44.2%) health care personnel, 395 (22.4%) first responders, and 590 (33.4%) other essential workers. Among health care personnel, 49.2% worked in inpatient, 41.9% in outpatient, and 9.0% in residential health care settings. Among first responders, 6.3% worked as correctional officers, 35.9% as fire fighters, 35.9% in law enforcement, and 21.8% in nonfire EMS. Among other essential workers, 76.7% worked in education, childcare or social support services, 9.2% in retail or hospitality, and 14.1% in other essential infrastructure or operations.

Sex distributions differed by occupation, with only 37.0% of first responders being female vs 69.7% and 68.3% of health care personnel and other essential workers, respectively. Income distributions also differed by occupation with 25.0% of health care personnel reporting annual household incomes in the highest category measured (≥$200 000); only 5.8% of first responders and 6.9% of other essential workers reported incomes in this category (Table 1).

The crude incidence of SARS-CoV-2 infection was 13.2 and 7.4 infections per 1000 person-weeks at risk for first responders and other essential workers, respectively, compared with 6.7 infections per 1000 person-weeks for health care personnel (Figure). For health care personnel subcategories, incidence was 6.1 per 1000 person-weeks in outpatient settings, 6.9 in residential settings (eg, long-term care), and 7.1 in inpatient settings. For first responder subcategories, incidence per 1000 person-weeks at-risk was elevated compared with health care personnel for every subgroup including corrections (16.0), fire service (12.9), law enforcement (12.2), and nonfire EMS (14.0) personnel. For other essential worker subcategories, incidence per 1000 person-weeks was higher for individuals in education, childcare or social work (10.1), and essential infrastructure (10.3), and lower among workers in retail or hospitality (7.0) and essential operations (4.7).

**Figure.**
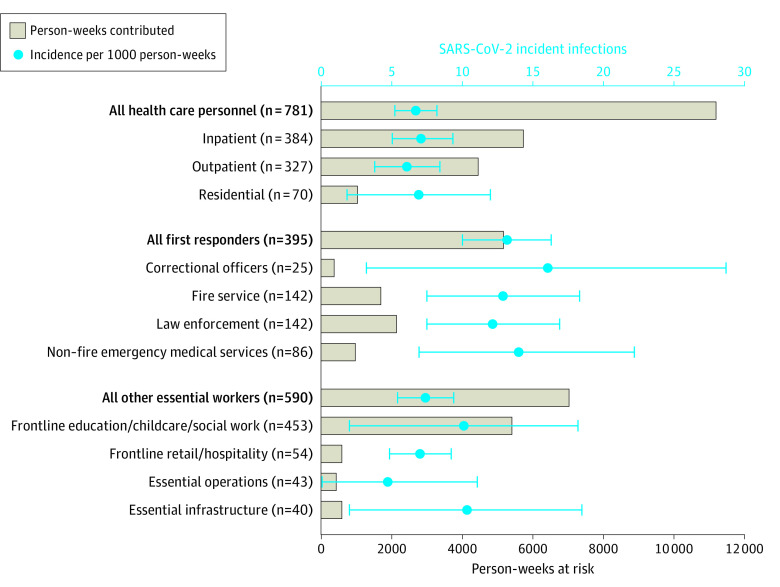
Person-Weeks Contributed and Crude Incidence of SARS-CoV-2 Infection per 1000 Person-Weeks at Risk for AZ HEROES Participants by Occupation^a^ AZ HEROES denotes the Arizona Healthcare, Emergency Response, and Other Essential Workers Study. ^a^Essential operations includes workers in sectors requiring in-person work (eg, post office or 911 call centers) and essential infrastructure includes workers in utility and municipal services (eg, waste management, electricity, water).

In unadjusted negative binomial regression models, first responders had a higher risk of SARS-CoV-2 infection than did health care personnel (IRR, 2.01; 95% CI, 1.44-2.79). In the adjusted LASSO-optimized model, the result was somewhat attenuated for first responders (IRR, 1.60; 95% CI, 1.07-2.38). Age was an independent risk factor; every 10-year increase in age was associated with a 19% reduction in risk in adjusted LASSO-optimized models (IRR, 0.81; 95% CI, 0.70-0.95). Risk of infection among other essential workers was no different than among health care personnel in either unadjusted or adjusted models (Table 2).

## Discussion

In this prospective cohort of Arizona workers, first responders had higher incidence of SARS-CoV-2 infection than health care personnel. Individuals were only categorized as health care personnel if their work involved direct patient care. Therefore, both health care personnel and first responders in this study had the potential for frequent occupational exposure to SARS-CoV-2. Recent studies^6^ suggest that occupational exposure has been reduced in health care personnel owing to improved infection prevention in health care settings. Equivalent improvements may not have penetrated first responder communities. Other possibilities that might have driven higher infection rates among first responders were exposures that were unaccounted for within the work setting (eg, eating, exercising, travel to/from scene) or exposures outside of the work setting (eg, mitigation efforts within household or private gatherings). Further elucidation of the causal mechanisms driving higher infection rates is necessary to determine which mitigation efforts should be prioritized for first responders.

Federal agencies have acknowledged the nexus between workers and communities as critical to perpetuating or preventing transmission of SARS-CoV-2.^13^ Tailored guidance for first responders who typically work long shifts in the presence of coworkers, including sleeping in close quarters, may be needed. For example, guidance from the US Centers for Disease Control and Prevention^14^ for EMS personnel recommends that workers wear a mask in any space where they may encounter coworkers. This recommendation likely needs to be contextualized to account for long shifts where coworkers drive, eat, and sleep in close quarters. As evidence substantiating the importance of ventilation has emerged, practical guidance for assessing and improving ventilation in workplace settings may be needed. Finally, addressing the elevated infection risk in first responders from a policy perspective should consider multiple mitigation targets, including vaccination, PPE provision and use, and development of educational materials.

In multivariate models, increasing age was shown to be associated with lower incidence of infection. For every 10-year increase in age, model-predicted incidence of SARS-CoV-2 infection decreased by 19%. This is consistent with COVID-19 case trends in Arizona during the study period, when rates of infection among adults were highest for those less than 24 years and lowest for those more than 64 years of age.^15^

### Limitations

This study was subject to several limitations. First, because the study was confined to Arizona workers, findings may not be generalizable to other states. Of note, 23% of study participants were Hispanic, which falls between the national (18%) and Arizona (32%) population proportions.^16^ Sex distributions within the AZ HEROES cohort tracked broadly with US Bureau of Labor Statistics data, which shows females compose the majority (74%) of health care practitioners (70% of health care personnel in the AZ HEROES cohort were female). Similarly, the Bureau of Labor Statistics reports that females compose the minority (23.6%) of those working in protective services (ie, first responders); 37.0% of first responders in AZ HEROES were female.^17^ Findings may not be generalizable to all health care workers because we excluded those who did not provide direct patient care (eg, administrators, housekeepers).

Second, broad occupational categories preclude precise estimates of occupational risk for specific occupation types. Despite the broad confidence intervals in the Figure, we note elevated rates for all first responder subcategories, indicating that their elevated risk was not driven by 1 particular occupational subcategory. Among first responders, incidence of SARS-CoV-2 infection ranged from 12.2 per 1000 person-weeks for law enforcement to 16.0 for correctional officers. Similarly, for health care personnel, incidence did not vary widely by occupational subcategory; the lowest incidence among health care personnel occurred among those working in outpatient settings and the highest occurred among those working in inpatient settings, 6.1 vs 7.1 per 1000 person-weeks, respectively.

Third, the finding that other essential workers were not at elevated risk compared with health care personnel could be biased by the high proportion of educators in this category. Many of these workers enrolled while schools were open but periodic school closures may have limited their contact with others during the observation window.^18^ Finally, health care personnel were recruited earlier than first responders and other essential workers, and thus findings could be biased by their having more cumulative person-weeks at-risk during periods of lower community incidence. However, participant-specific county-level exposure was included in adjusted models, which accounted for aggregate county-level exposure for each participant’s duration in the study.

## Conclusions

In this prospective cohort study of Arizona essential workers, prevaccination incidence of SARS-CoV-2 infection among first responders was twice as high as among health care personnel in unadjusted models, and 60% higher after adjusting for potential confounders. Incidence of SARS-CoV-2 infection in other essential workers was similar to that of health care personnel in this cohort. These findings support greater prioritization of first responders as a high-risk group in the context of the COVID-19 pandemic.
